# Effects of proprioception and core stability training on gait parameters of deaf adolescents: a randomized controlled trial

**DOI:** 10.1038/s41598-023-49335-3

**Published:** 2023-12-10

**Authors:** Hamed Zarei, Ali Asghar Norasteh, Lauren J. Lieberman, Michael W. Ertel, Ali Brian

**Affiliations:** 1 Corrective Exercises and Sports Injury Department, College of Physical Education & Sport Sciences, Faculty of Physical Education & Sport Sciences, University of Guilan, kilometers 10 Rasht-Ghazvin Road, Rasht, 4199613776 Iran; 2 Physiotherapy Department, Faculty of Medicine, Guilan University of Medical Sciences, Rasht, 4199613776 Iran; 3 Department of Kinesiology, Sport Studies and Physical Education, State University of New York (SUNY), Brockport, NY 14420 USA; 4 Department of Physical Education, University of South Carolina, Columbia, SC USA

**Keywords:** Health care, Medical research

## Abstract

The current study aimed to explore the effects of proprioception versus core stability training over 8 weeks on the gait parameters of deaf adolescents. A total of 20 deaf adolescents were randomized into two groups: one group receiving proprioception training (PT, n = 10), another group receiving core stability training (CST, n = 10), and eleven typically developing adolescents assigned into the control group (CON; n = 11). Gait was recorded by two digital cameras; then, using the Kinovea software, the parameters of gait included: gait velocity, cadence, stride length, stride time, stance time, and swing time were calculated in terms of percentages of the walking cycle. After 8 weeks of PT, no significant differences were observed for all gait parameters between PT and control groups (*p* > 0.05). Also, after 8 weeks of CST, no significant differences were observed in gait velocity and cadence between the CST and control groups (*p* > 0.05). However, after 8 weeks of CST, stride length (*p* = 0.02) was higher in the control group; Stride time (*p* = 0.03), stance time (*p* = 0.04) and swing time (*p* = 0.04) were higher in the CST group. Moreover, after 8 weeks of PT, values showed significant improvements in all gait parameters (*p* = 0.001). Also, after 8 weeks of CST, values showed significant improvements in gait velocity and cadence (*p* = 0.001), but no significant differences were observed in other gait parameters (*p* > 0.05). The findings of this study indicated that PT improved all gait parameters, whereas CST improved gait velocity and cadence. The results of the present study also demonstrated that PT had a greater effect on gait parameters of deaf adolescents compared with CST. It seems that PT induces more training effects than CTS for enhancing gait parameters of deaf adolescents.

**Trial registration**: Clinical trial registry number: IRCT20170312033029N2. URL: https://en.irct.ir/trial/25584.

## Introduction

Deaf is associated with the loss of labyrinth function and may contribute to postural instability^1^. This balance disorder resulting from vestibular dysfunction due to damage to the inner ear can negatively affect the development of movement skills and cause more motor disorders for deaf children and adolescents when compared with typically developing peers^2^. However, it is necessary to examine the contribution of this system in gait control of deaf adolescents. Previous studies on the motor function of deaf children and adolescents demonstrate shorter and more irregular steps^3^, slower gait speed^4^, more cautious gait^3^, and decreased performance in gait-related functional tasks^5^. However, deaf children and adolescents have greater stride cycle duration^6^, a different frequency content of gait ground reaction forces^7^, and more electromyographic activity in the tibialis anterior muscle, gastrocnemius muscle, and vastus lateralis muscle during walking, as compared to their peers with typically development^8^.

Previous results also indicate that the values for the stance and swing phases during gait were significantly higher in deaf children and adolescents^6^. As a result, cycle duration was also higher in deaf children and adolescents, which was a speed barrier^6^. The speed changes in the pattern of transitional movements affect muscle activation patterns, muscle fiber length^9^, and specific stepping parameters^10^. In addition, higher gait ground reaction forces in deaf children and adolescents can be associated with more load in the proximal region of the joint^11^. The worse gait performance of deaf children and adolescents gait parameters may be triggered by balance disorders, given the evidence that balance is an unpredictable motor skill for the execution of gait^5,12^.

Given the results of these studies, deaf children and adolescents can suffer neuromotor developmental delays and problems with certain motor abilities. Additionally, these individuals may complain of discomfort during games and physical activities typical of their age. Most importantly, deaf children and adolescents may have difficulties keeping up with other individuals of the same age and sex and interacting with their peers^13^. These findings emphasize the need to develop prevention programs and appropriate therapeutic measures in the school environment to correct and/or improve motor performance and gait of deaf adolescents. Consequently, the quality of life of school deaf children may be impacted, thereby preventing falls and more serious morbidities in this population.

Proprioception training programs seem to restore motion control by maximizing sensory input from different parts of the body to improve motor control^14,15^. In addition, core stability training is a modality of training that focuses on trunk muscles. Enhancing trunk muscle forces provides stability in the core body, leading to postural performance improvements^16–19^. Therefore, this study aimed to examine the effects of proprioception and core stability training on gait parameters of deaf adolescents. In this study, we have two primary aims; (1) To determine the effects of 8 weeks of proprioception training and core stability training on gait parameters of deaf adolescents and (2) To compare adaptive changes in gait parameters between the two training modalities after 8 weeks of the intervention protocol.

## Methods

### Participants

Deaf adolescents from a regional deaf center within a local deaf school (congenital sensorineural type deafness-with unilateral or bilateral hearing aids or hearing loss) and typically developing adolescents (from the local typically developing school) volunteered to participate in the study. deaf adolescents were assigned into two groups including the proprioception training group (PT; *n* = 10) and the core stability training group (CST; *n* = 10). Typically developing adolescents were assigned to the control group (CON; n = 11). The sample size was calculated based on a previous study by Baluchi et al.^20^ with an alpha level of 0.05 and an actual power (1-beta) of 0.80. The sample size calculation was considered a power calculation to detect between-group differences in gait velocity. The analysis (G × Power, Version 3.1.9.2, University of Kiel, Germany) revealed that a sample size of *N* = 10 would be sufficient for each group to detect the effect of the training intervention on gait parameters of deaf male adolescents.

The inclusion criteria for the study included: (1) male adolescents (Due to environmental conditions, female participants were not available), (2) hearing range greater than 75 decibels (This criterion was intended only for deaf adolescents), (3) skeletal maturity which assessed by using Tanner Whitehouse method^21^, and (4) age range of 15–18, (5) Not participating in any professional sports and only participating in the general physical education classes of the school. The exclusion criteria for the study included, (1) use of neurological drugs that influence gait parameters, (2) history of lower extremity injuries within the last 6 months, (3) history of muscular/neural ailments (myopathy, myositis, peripheral neuropathy, muscular dystrophy), (4) postural abnormality in the upper or lower extremities (such as kyphosis, lordosis, forward head, knee valgus and knee varus which was measured by the New York Posture Rating Chart^22^), (5) surgery or fracture within a year before the study, (6) insulin-dependent diabetes, (7) joint rheumatoid arthritis, (8) diagnosed cerebrovascular disease or any other disease that interferes with sensory input, (9) lower extremity rotational deformities (increased anteversion, tibial torsion) or pes planovalgus. Before the initiation of the study, the adolescents and their parents were informed about the research procedures, and their written consent was obtained.

Because of environmental and cultural conditions, this study did not evaluate the vestibular function of the deaf adolescents. However, there is a standardization of deaf children and adolescents in relation to the degrees of hearing loss. The authors selected deaf adolescents with the highest degrees of hearing loss (hearing range greater than 75 decibels) and the evidence demonstrates that, the greater the degrees of hearing loss, the worse the balance of deaf children and adolescents^1,23–25^ and the greater their chances of presenting vestibular dysfunction^25–28^. Therefore, the authors tried to choose the deaf adolescents with the worst performance to rehabilitate them.

**Figure 1:**
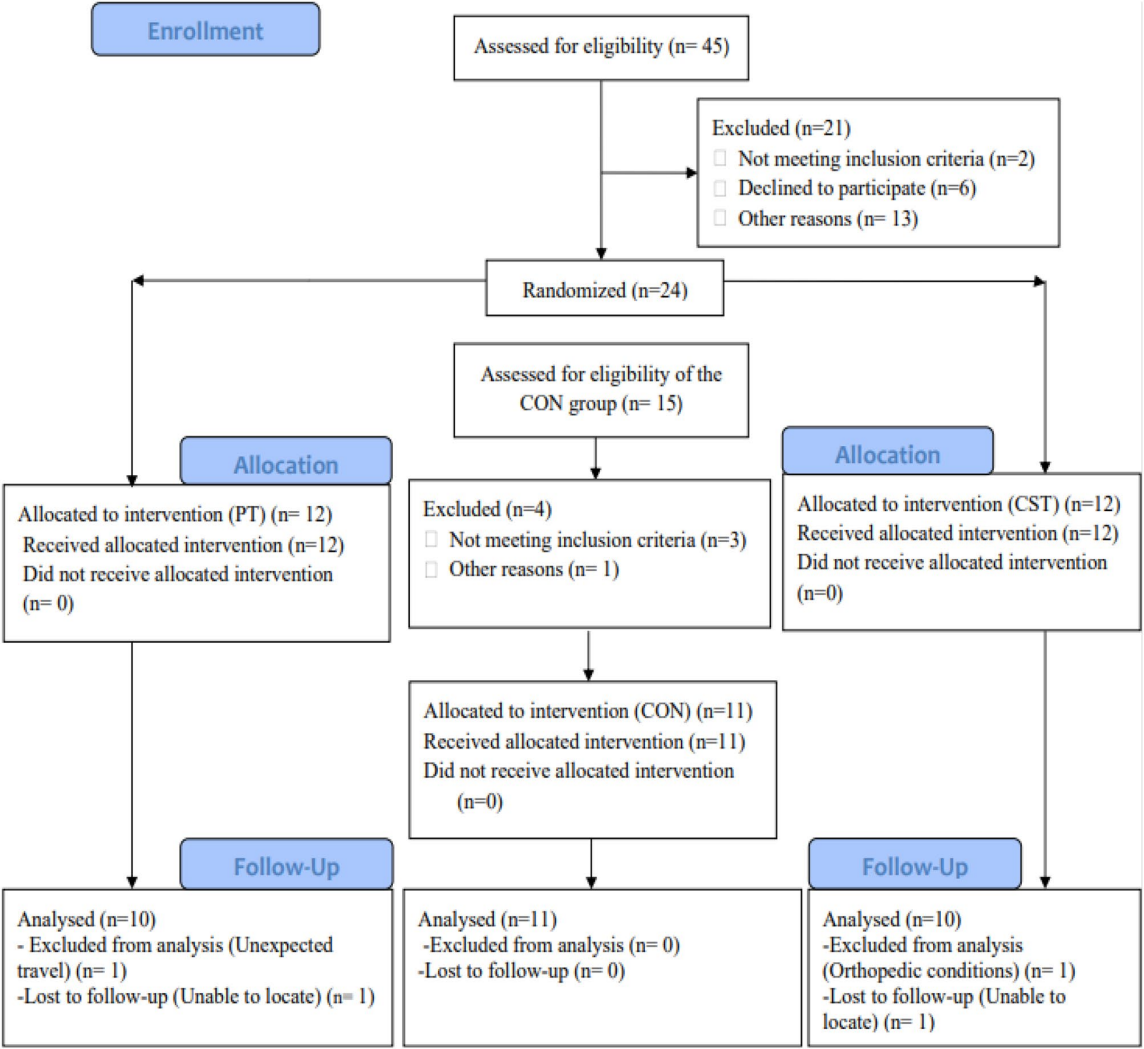
Flowchart of eligibility, inclusion and exclusion criteria, and analysis. PT: proprioception training, CST; core stability training, CON; control.

In the primary stage of the study, 45 deaf male adolescents were recruited in the study. Based on inclusion and exclusion criteria, 24 participants were included and allocated into two intervention (PT and CST training) groups (*n* = 12 for each group). Based on inclusion and exclusion criteria 11 participants were allocated into the control group. To be included in the final analyses, the participation of intervention groups was required to complete all the training sessions and attend all assessment sessions. As a result, four participants in the intervention groups were excluded from the study, and 10 participants from each intervention group and 11 participants from the control group were included in the final analysis (Fig. 1).

### Study design

This study followed a longitudinal randomized control trial with convenience sampling. This trial followed the CONSORT 2010 checklist of information to include when reporting a randomized trial^29^. Adolescents were pair matched according to their hearing range and then divided into two experimental groups. Quadruple block randomization was performed using computer software generating a table of numbers by a blinded member of the study team. PT, for the proprioception program, was performed for an hour per day, 3 days per week. CST, for the core exercises, were performed totaled an hour per day, 3 days per week. Finally, the control group did not perform any exercise or training program. The study period lasted 11 weeks by adhering to a strict as follows: 1st week—familiarization period; 2nd week—pretest period; 3rd to 10th week—training period; 11th week—post-test period. Two weeks before the initiation of the training period, a researcher and physiotherapist communicated with the participants and standardized the training procedures. Three sessions of weekly exercise were co-created between the trainer and participants. Participants were instructed to run the practices properly. All directions were explained to each participant via total communication, body language, and sign language involving speech and demonstration.

This study was registered and allocated by the Clinical Trials (IRCT20170312033029N2) and was approved by the Institute of Physical Education and Sport Sciences (Iran) IR.SSRI.REC.2017 146; and all experiments were performed in accordance with relevant guidelines and regulations.

### Procedures

The adolescents underwent 2 days of testing, including pre and post-tests. They were tested exactly at the same time of day (10 to 12 A.M.), same time of training hour, and same day of the week as the pre-test day to minimize the effect of circadian variations in the test results at the school gym.

### Measurements

Height was measured by a stadiometer (Seca 222, Terre Haute, IN) mounted on the wall and recorded to the nearest 0.5 cm^30^. Body mass was measured to the nearest 0.1 kg using a digital scale (Tanita, BC-418MA, Tokyo, Japan)^30^. Body mass index (BMI) was calculated (kg/m^2^).

The geometric figure was recorded using two CASIO Exilim EX-ZR700 high-definition video cameras with the following setup: resolution: 1280 × 720 pixels per inch; frequency: 30 Hz; focal length: 52 mm; sensitivity: ISO 400; aperture: 2.7; shutter. The lens was located at a height of 0.68 m from the ground, and 30 frames per second were used for the recording of gait. These cameras were placed on both sides of the walking path at a different distance of two meters from each other^31^. A total of 10 retroreflective markers with a diameter of 14 mm were positioned on the skin overlying specific bony landmarks or anatomical positions of the lower body. Markers were placed on the anterior superior iliac spine, greater trochanter, external condyle of the knee, lateral malleolus, and fifth metatarsal of both limbs^32^. Participants were then asked to walk barefoot a distance of six meters at the desired speed (self-selected comfortable gait speed) to record their gait information. To ensure proper and natural gait, participants were asked to make several attempts at the desired speed before taking the main test. Despite careful measurement, some trials had to be omitted due to irregularities in the kinematics. Each participant was asked to walk six meters in five trials with arbitrary speed. In each trial, one stride (A stride was chosen in which the markers and limbs could be seen well and more detailed information could be extracted from the parameters) was selected for analysis. In total, five strides were examined for each participant. The final score in each parameter for each participant was divided by five and the final score of each participant was entered into the SPSS software. The video was framed by Kinovea software (version 0.8.15) The criterion validity between Kinovea ® and VICON Motion System ® is excellent reported for the gait parameters (r > 0.80)^33^. For the spatiotemporal analysis, the right lower limb was assessed. The variables examined in this study were cadence (60/time between two successive heel strikes of one limb and the other limb), stride length (distance between two successive heel strikes of the same limb), stride time (time between two successive heel strikes of the same limb), stance time (time between heel strike to toe-off of the same limb), and swing time (time between toe-off to heel strike of the same limb).

Two marks were placed on the footbridge that the participants had to walk on. These marks were placed two meters between them, to contrast the measurement in the video and to obtain the parameter of stride length. The procedure for obtaining the spatiotemporal parameters began with the drawing of a line joining the two marks located on the walkway. Next, we contrasted its measurement in the program (2 m), which allowed us to obtain the stride length with the use of the “line” tool. To obtain the step time, stride time, stance time, and swing time parameters, the “chronometer” tool in the software was used. If it is activated from the initial contact of a foot to the initial contact of the contralateral, we will obtain the step time to calculate cadence. Additionally, if the chronometer is activated from heel strike to toe-off the same limb the stance time will be obtained for individuals. Finally, if measured from the toe-off to heel strike of the same limb, individuals will be measured through the swing time^33^. In addition, three measurements were made in 10 participants 24 h apart to determine the inter-rater reliability of the test. The intraclass correlation coefficient (ICC) for this test was 0.90, which is considered high.

To measure gait speed, a six-meter walking speed test was used^34^. In this test, participants were asked to walk a distance of six meters at a maximum speed. Each participant performed this test twice and the best record was calculated. Gait speed (m/s) was obtained by dividing distance by time. To measure the distance more accurately, we also considered two meters before and after the main distance (6-m distance) so that the increase and decrease in gait speed at the beginning and end of the path may not affect the test accuracy. The intrarater ICC for this test was 0.97.

### Interventions

Each training session for the PT and CST groups lasted 60 min, including five minutes of warm-up, 50 min of main training (i.e., PT or CST), and five minutes of cooling down. The training program for the PT group was applied regarding Martínez-Amat et al.^35^, Clark and Burden^36^ and Carmeli et al.^37^ recommendations. The adolescents performed the protocol with open eyes but then wore a blindfold to perform the training exercises. To standardize the training procedures, three sessions of weekly exercises were appointed for participants to get to know the trainer. Participants were instructed to run the practices properly. Among the exercise protocols, the exercises that were suitable for the participants and the participants could perform them were selected. Some exercises used in this study were on the tilt board, wobble board, and boss balls. Considering the instability of the support surface, individuals preferred the hip strategy rather than the ankle strategy. In the hip strategy, more proximal joints are engaged first. The body provides appropriate stability for the optimal balance of proximal joints and consequently improves the proprioception of joints. Also, PT is performed in a closed kinetic chain and causes muscle contraction, and makes mechanoreceptors in the skin, joints, and capsules work more effectively. Thus, the efficiency of proprioceptive receptors is promoted. The details of the selected exercises are provided in Tables 1 and 2.

**Table 1:** Proprioception training exercises.

Exercises number	Instructions
1	The subject sits between parallel bars on Swiss balls. With upright trunk, he performs knee flexion and extension
2	The subject sits between parallel bars and moves the tilt board forward and backward
3	The subject sits between parallel bars and moves the tilt board sideways
4	The subject stands between parallel bars on tilt board and maintains his balance for 15 s
5	The subject moves between parallel bars on buso balls with alternative legs
6	A repetition of exercises 2, 3, and 4 with a bent knee
7	The subject stands with the dominant leg on wobble board and maintains his balance for 15 s
8	The subject stands between parallel bars on buso balls for 15 s
9	The subject stands with the dominant leg on buso balls and maintains his balance for 15 s
10	A repetition of exercises 7, 8, and 9 with a bent knee
11	The subject lies down supine with legs on a Swiss ball, moving up his hip and back and maintains balance with his hands
12	Exercise 11 is repeated with hands on stomach
13	The subject leans against a Swiss ball on the wall. With an upright trunk, he bends his knees a little and returns back to his initial position
14	Exercise 13 is repeated with knees bent 90°
15	Walk forward, walk on heels, tendon gate, sideway walk, walking on toes (2 sets of 15 repetition on a 10 m path)
16	Exercise 15 is repeated on a rough path

Time-oriented exercises are performed in 2 sets of 15 s with 1 min rest between each set.

Repetition-oriented exercises are performed in 2 sets of 10–15 repetition with 1 min rest between each set.

**Table 2:** Progressive plan of proprioception training exercises.

Weeks	Exercises development
Week 1	Performing exercises 1–6 putting both hands on parallel bars to maintain balance
Week 2	Performing exercises 1–6 putting one hand on parallel bars to maintain balance
Week 3	Performing exercises 1–6 with no use of parallel bars
Week 4	Performing exercises 7–14 putting both hands on parallel bars to maintain balance (exercises 11–14 without use of parallel bars)
Week 5	Performing exercises 7–14 putting one hand on parallel bars to maintain balance (exercises 11–14 without use of parallel bars)
Week 6	Performing exercises 4–14 with no use of parallel bars
Week 7	Performing exercise 15
Week 8	Performing exercise 16

The training program for the CST group was applied using Jeffreys^38^ recommendations at three levels. At level one, the participants performed exercises in (a) a stable environment and (b) slow movements in an unstable environment. At level two, the participants performed exercises in a stable environment followed by dynamic movements in a stable environment. Finally, in level three, resisted dynamic movements in an unstable environment are performed. Medicine balls, Swiss balls, and an athlete’s body weight were used. The details of the exercises are provided in Table 3. The control group did not perform any exercise and training programs, participating in the general physical education classes of the school. Both training loads increased progressively throughout the study. The amount of training progress was the same for everyone. The participants who did not progress in the exercises, as well as their groupmates, were given more time for their training to reach their groups in terms of the progress of the training. Each participant was supervised and verbally encouraged to achieve proper technique of each exercise by a researcher and physiotherapist to control all the training procedures and tests. Both trainings were conducted by both a physiotherapist and a researcher who had sufficient skills and specialized certificates in the field of these trainings. Each training session was supervised by a coach and physiotherapist. Each training session had a trainer-to-trainee ratio of 1:5.

**Table 3:** Exercises program for core stability.

Weeks	Exercises	Sets × reps
Week 1and 2	Holding the abdomen in. in a supine position	3 × 20
	Holding the abdomen in. in a prone position	3 × 20
	Holding the abdomen in. in a squatting position	3 × 20
Week 3	Holding the abdomen in. with one leg in the abdomen in a supine position	3 × 20
	Holding the abdomen in. with one leg in the abdomen in a prone position	3 × 20
	Side lying bridge	6 × 10 s hold
Week 4	Holding the abdomen in, in supine position with limbs up and keep hands and feet close together	3 × 20
	Squatting with raising one leg out of back	3 × 20
	Trunk rotation while holding weights in each hand	3 × 20
Week 5	Sitting on a Swiss ball and holding the abdomen in	3 × 10 s
	Squatting while the Swiss ball is on the shoulder	3 × 15
	Trunk rotation while holding weights in each hand	3 × 10
Week 6	Doing Lunge in a 45° inclined direction to the left or right	3 × 12
	Bridge (shoulder and hands on the ground and the bringing up on hip and foot)	3 × 10 s hold
Week 7	Lying supine on the Swiss ball and rotating the trunk to the sides	3 × 15
	Doing the above exercise with holding weights in the hand	3 × 15
	Side lying bridge with bringing up the leg	6 × 10 s hold
Week 8	Lying supine on the Swiss ball and holding the abdomen in and bringing one leg up	3 × 20
	Raising the opposite arm and leg while squatting	3 × 20
	Bridge so that the feet are placed on the Swiss ball and raise one foot	3 × 15 s hold

### Statistical analysis

The normality of all data before and after interventions was checked with the Shapiro–Wilk test and data are presented as mean ± standard deviations (SDs). In addition, data were analyzed by 2-way [3 (group) × 2 (time)] repeated-measures analysis of variance (ANOVA) using the SPSS statistical software package (SPSS version 21.0 for Windows, SPSS Inc., Chicago, IL, United States) to determine differences between the groups in the gait parameters at pre and post-training. When a significant *F* value was achieved, a Bonferroni post hoc test was used to detect differences in the measures. Cohen's d^39^ was also used to calculate the effects of training (effect size [ESs]). Threshold values for assessing the magnitudes of ES were < 0.2, trivial; 0.2–0.6, small; 0.6–1.2, moderate; 1.2–2.0, large; 2.0–4.0, very large; and > 4.0, perfect^40^. The effect size is reported with a 95% confidence interval (CI) for all analyzed measures. The significance level was set at p ≤ 0.05 and the power (1-beta) was 0.80.

## Results

The characteristic information of the participants is presented in Table 4. No significant difference was found between groups in characteristic information (*p* > 0.05) (Table 4).

**Table 4:** Baseline measurements (mean ± SD).

Characteristic	Proprioception training (n = 10)	Core stability training (n = 10)	Control group (n = 11)	Total group (n = 31)	p-value
	Mean ± SD	Mean ± SD	Mean ± SD	Mean ± SD	
Age (y)	16.4 ± 1.1	16.9 ± 0.9	17.1 ± 1.4	16.8 ± 1.0	0.42
Height (cm)	165.7 ± 5.3	166.8 ± 5.1	169.9 ± 6.3	165.4 ± 5.2	0.31
Body mass (kg)	61.2 ± 5.1	62.2 ± 5.3	60.7 ± 4.9	61.8 ± 5.2	0.64
BMI (kg/m^2^)	22.3 ± 2.4	22.3 ± 2.2	22.4 ± 2.7	22.2 ± 2.3	0.23
HL (dB)	80.6 ± 4.1	81.1 ± 4.8	– ± –	80.85 ± 4.4	0.46

BMI: body mass index; HL: hearing loss.

### Comparisons gait variables between intervention and control groups in the pre-test.

At the pre-test, no significant differences were observed for gait parameters between intervention groups (*p* > 0.05). However, gait velocity (*p* = 0.01), cadence (*p* = 0.02) and stride length (*p* = 0.01) were higher in the control group as compared to the intervention groups. Stride time (*p* = 0.03), stance time (*p* = 0.04) and swing time (*p* = 0.02) were higher in the PT group as compared to the control group. Finally, stride time (*p* = 0.01), stance time (*p* = 0.02), and swing time (*p* = 0.03), were higher in the CST group as compared to the control group.

### Comparisons gait variables between intervention and control groups in the post-test

After 8 weeks of PT, no significant differences were observed for all gait parameters between PT and control groups (*p* > 0.05). The PT group showed a significant pre-to-post change in all gait parameters (*p* = 0.001) while the control group showed no change in gait parameters from pre-to-post (*p* > 0.05). Also, after 8 weeks of CST, no significant differences were observed in gait velocity and cadence between the CST and control groups (*p* > 0.05). However, after 8 weeks of CST, stride length (*p* = 0.02) was higher in the control group as compared to the CST group. Stride time (*p* = 0.03), stance time (*p* = 0.04) and swing time (*p* = 0.04) were higher in the CST group as compared to the control group.

### Comparisons gait variables between PT group and CST group in the post-test

Furthermore, after 8 weeks of training, no significant differences were observed in gait velocity and cadence between PT and CST (*p* > 0.05). Conversely, stride length (p = 0.04) was higher after 8 weeks of training in the PT group as compared to the CST group. Stride time (*p* = 0.04), stance time (*p* = 0.03), and swing time (*p* = 0.02) were higher in the CST group as compared to the PT group (Table 5).

**Table 5:** Gait parameters change in the groups [n = 31].

Conditions	Groups	Time point values (mean ± SD)			Effect size (95% CI) and p value	
		Pre	Post		Pre to post	p-value
The walking speed test						
Gait velocity (m/s)	PT	1.2 ± 0.07*	1.5 ± 0.04^†^	G = 0.21	2.46 (0.21 to 2.44)^e^	0.001
	CST	1.2 ± 0.04*	1.5 ± 0.06^†^	T = 0.001	1.29 (0.47 to 1.49)^d^	0.001
	CON	1.6 ± 0.08	1.6 ± 0.05	G × T = 0.02	–	
Self-selected gait speed						
Cadence (steps/min)	PT	72.8 ± 2.1*	90.2 ± 3.21^†^	G = 0.26	2.12 (0.07 to 1.93)^e^	0.001
	CST	73.4 ± 2.14*	89.9 ± 3.42^†^	T = 0.001	1.91 (0.01 to 1.84)^d^	0.001
	CON	96.1 ± 5.12	92.1 ± 4.17	G × T = 0.03	–	
Stride length (cm)	PT	115.2 ± 3.1*	119.3 ± 4.70^†^^✯^	G = 0.48	1.89 (0.29 to 2.18)^d^	0.001
	CST	116.3 ± 4.1*	117.1 ± 3.1*^✯^	T = 0.001	0.32 (0.41 to 0.93)^b^	0.29
	CON	120.3 ± 4.5	119.5 ± 2.71	G × T = 0.04		
Stride time (s)	PT	1.59 ± 0.41*	1.17 ± 0.32^†^^✯^	G = 0.31	2.08 (0.21 to 1.87)^e^	0.001
	CST	1.6 ± 0.41*	1.41 ± 0.37*^✯^	T = 0.001	0.18 (0.31 to 0.63)^a^	0.19
	CON	1.26 ± 0.23	1.31 ± 0.45	G × T = 0.01		
Stance time (s)	PT	0.67 ± 0.05*	0.57 ± 0.04^†^^✯^	G = 0.26	1.96 (0.32 to 2.12)^d^	0.001
	CST	0.66 ± 0.04*	0.64 ± 0.02*^✯^	T = 0.001	0.21 (0.12 to 0.55)^b^	0.21
	CON	0.57 ± 0.02	0.56 ± 0.05	G × T = 0.02		
Swing time (s)	PT	0.41 ± 0.02*	0.36 ± 0.03^†^^✯^	G = 0.51	2.02 (0.24 to 2.34)^e^	0.001
	CST	0.42 ± 0.03*	0.41 ± 0.03*^✯^	T = 0.001	0.12 (0.06 to 0.21)^a^	0.36
	CON	0.34 ± 0.02	0.35 ± 0.03	G × T = 0.03		

Values are mean ± SD.

cm: centimeter; sec: second; m/s: meters per second; steps/min: steps per minute; PT: proprioception training; CST: core stability training; CON: control.

*Significant differences compared to CON (p < 0.05).

^†^Significant differences compared to pre (p < 0.05).

^✯^Significant differences between PT and CST (p < 0.05). G; group, T; time. CI; confidence interval. a, trivial; b, small; c, moderate; d, large; e, very large ES.

## Discussion

This study was designed to examine the effects of an 8-week PT and CST on gait parameters of deaf adolescents. The findings of this study indicated that the PT group improved all gait parameters at post-training intervention. The results also demonstrated that 8 weeks of CST improved some gait parameters such as gait speed and cadence but not other parameters such as stride length, stride time, stance time, and swing time. In this study, two digital cameras and Kinovea software were used to analyze each of the gait parameters as it is asserted in the literature to be a reliable software for measuring gait parameters^41^.

The results of the present study showed a significant difference between of deaf adolescents and typically developing adolescents in terms of gait parameters in the pre-test. Uysal et al.^42^ supported that these differences are attributed to typically developing individuals' reliance on sensory information. As a result, these individuals have longer stride lengths and faster gait speeds than people with sensory impairments. Therefore, one of the reasons for the effectiveness of PT on gait parameters of deaf adolescents in this study can be an increase in information of proprioceptor receptors. The present results are inconsistent with Majlesi et al.^4^ which showed that a 12-session balance training intervention has a significant effect on the balance of deaf children, but does not improve the gait speed. The inconsistency may be due to the duration of training sessions since PT was performed during 24 sessions in the present study; While PT was performed in 12 sessions in the previous study^4^.

Consistent with the present study, which showed that 8-week PT improves the gait parameters of deaf adolescents, the results of previous studies have shown that balance training or PT has a positive effect on the gait parameters^43,44^. Also, Hussein et al.^45^ investigated the effect of simultaneous proprioceptive-visual feedback on gait of children with spastic diplegic cerebral palsy. The results of study showed that simultaneous proprioceptive—visual training might improve spatial and temporal gait parameters with no effect on kinetic gait parameters of children with spastic diplegic cerebral palsy. Furthermore, in line with the effect of CST on gait parameters, Chung et al.^46^ investigated the effects of CST on dynamic balance and gait function in stroke patient. The results of study showed that CST improve balance but improve some gait parameters. However, Li and Xie^47^ showed that CST improve all gait parameters. Moreno-Segura et al.^48^ in a systematic review and meta-analysis study, showed that CST improves trunk function and balance in acute and chronic stroke patients, but no changes were found in gait parameters. The results regarding the effect of CST on gait parameters are contradictory and more studies are needed to draw better conclusions.

PT exercises have led to improved and more organized functions of the mechanoreceptors, thus facilitating and improving the efficiency of proprioceptors^49^. As a result, PT exercises to improve proprioception and motor function across a range healthy and clinical populations^50^. Also, an important aspect of this set of exercises was using tilt boards, wobble boards, and boss balls. In this case, the person uses the hip strategy instead of the ankle strategy because of the instability of the level of support. Therefore, the hip strategy can be applied^51^. The hip strategy is typically used to control body posture when larger disruptive forces exert the torque required to restore balance^51^. In these exercises, more proximal joints become active first, due to the instability of the level of support^52^. With these exercises, the body has provided good stability and optimal balance in the proximal joints, thus improving proprioception in all lower limb joints.

The results of the present study also showed that the PT group had a larger effect on the gait parameters of deaf adolescents compared with the CST group. Also, all gait parameters improved in the PT group, while some gait parameters changed in the CST group. This showed that PT had a greater effect on the gait parameters among deaf adolescents. By increasing the mechanoreceptors and neuromuscular coordination^49^, it seems that PT allows the central nervous system to activate the motor nerves of the muscles in a specific and coordinated pattern. As a result, PT increases the body awareness^53^, which in turn helps the person to perform functional activities more accurately and quickly because of increased balance and motor coordination^14^. Conversely, CST targets specific muscles, focuses on a specific area of ​​the body, and focuses on the control motor less frequently^54^, resulting in its lower effectiveness than PT.

Zhang et al.^55^, showed that vestibulospinal system (VOS) plays a fundamental role in providing balance and stability of the body in the gravitational field. The neural basis of plasticity and motor learning within the VOR pathways has been extensively examined^56,57^. Studies show the importance of head and body movements in vestibular rehabilitation and adaptation^58,59^. According to the physiological adaptations in skill learning, proprioceptive training can reduce the variability in the use of motor units, increase the plasticity of the motor cortex, or help people use their muscles to perform the motor task optimally^60^. It is interesting to note that recent evidence shows that proprioceptive training is related to increased corticospinal facilitation^61^; Therefore, it seems that the vestibular adaptations achieved by proprioceptive training remain for a long period of time. However, making better and more definite conclusions that require further studies and comparisons with other exercises.

### Limitations and future scope

This study has a few methodological limitations that should be disclosed. First, the findings from this study are specific to 15–18-year-old deaf male adolescents. More research is needed to identify whether our findings can be generalized to deaf female adolescents, in this age range. Second, the effects of PT can be compared with vestibular exercises; because both proprioceptive and vestibular training programs are sensory systems training programs, therefore, the comparison of these two types of training can be done to determine which training is more effective on gait parameters of deaf adolescents. Third, there was no blinding in the evaluation of the variables and blinding of evaluators; so, it is suggested that future researches consider this limitation. Fours, due to the lack of assessment of the vestibular function of the deaf adolescents, the results presented study do not allow us to conclude anything regarding the vestibular function of deaf adolescents. Finally, determining different times for retention after the completion of both training interventions could enhance our understanding of the effectiveness of training on gait parameters.

## Conclusion

The findings of this study revealed that PT improved all gait parameters at post-training intervention. The results of the present study also showed that 8-week CST improved some gait parameters such as gait speed and cadence. However, other parameters such as stride length, stride time, stance time, and swing time were not improved in this group. The results of the present study also demonstrated that PT had a greater effect on the gait parameters of deaf adolescents compared with CST. PT seems to have a better effect on the gait parameters of deaf adolescents as compared to CST.

## Data Availability

The datasets generated and analyzed during the current study are not publicly available due to privacy and restrictions, but are available from the corresponding author upon reasonable request.
